# Degradation of Electrical Performance in Transformer Oil Under Low-Temperature Moist Conditions and Its Microscopic Mechanisms

**DOI:** 10.3390/ma19214462

**Published:** 2026-10-23

**Authors:** Tonglei Wang, Tianxi Xie, Yuxing Chen, Zhengguang Chen, Qi Shi

**Affiliations:** 1 Electric Power Research Institute, State Grid Jiangsu Electric Power Co., Ltd., Nanjing 211103, China; spone@foxmail.com (T.X.); shiqi202605@163.com (Q.S.); 2 State Key Laboratory of Electric Insulation and Power Equipment, Xi’an Jiaotong University, Xi’an 710049, China; chenyuxing4013@163.com; 3 China Electric Power Research Institute, Beijing 100084, China; chenzhengguang@epri.sgcc.com.cn

**Keywords:** transformer oil, low temperature, moisture, conductivity, breakdown voltage, molecular dynamics, water clustering, interfacial polarization

## Abstract

Low-temperature environments pose challenges to transformer insulation reliability, as altered solubility and physical states of moisture in oil may trigger degradation mechanisms absent at ambient temperatures, yet the physicochemical processes remain poorly understood. This study combines macroscopic measurements with molecular dynamics (MD) simulations to investigate the bulk conductivity and alternating current (AC) breakdown characteristics of moist transformer oil across a wide temperature range. Conductivity and breakdown voltage were measured under controlled cooling conditions, while MD simulations tracked water cluster evolution and interfacial behavior at the molecular level. The results reveal a non-monotonic temperature dependence: conductivity exhibits a single-peak maximum near −5 °C, whereas breakdown strength reaches a pronounced minimum around −10 °C, with an offset between the two phenomena. MD simulations demonstrate that, as temperature decreases, water molecules transition from a dispersed state to nucleated aggregates and eventually form stable large-scale clusters. The low-temperature-induced nucleation and aggregation of moisture, which are much less pronounced under normal-temperature conditions, increase the oil–water interfacial area and enhance interfacial polarization, contributing to the anomalous increase in conductivity. At lower temperatures, stable water clusters act as dielectric “soft defects”, inducing local electric field distortion and thereby reducing the breakdown strength. The results establish a link between temperature-dependent moisture aggregation and macroscopic electrical degradation, providing a microscopic explanation for the anomalous temperature-dependent insulation behavior. These findings help to optimize insulation design and develop condition-assessment strategies for transformers in frigid regions.

## 1. Introduction

The reliable operation of modern power systems depends critically on transformers for voltage conversion and energy distribution, with transformer oil serving as both an insulating and cooling medium whose performance directly dictates equipment reliability and service life. During service, moisture inevitably invades the oil through breathing, aging, or maintenance processes, and even trace amounts of moisture can markedly reduce dielectric strength and accelerate the aging of oil-paper insulation because of its high polarity, making it a primary contributor to insulation failure [1]. Under low-temperature conditions, however, the solubility and physical state of moisture in oil undergo substantial changes, potentially triggering degradation mechanisms that are fundamentally different from those at ambient temperature, thus posing a particular threat to transformers installed in cold regions or subject to large thermal fluctuations [2,3,4,5]. Despite this practical importance, the underlying physicochemical processes remain poorly understood.

Previous studies have extensively documented that moisture increases dielectric loss and decreases breakdown voltage [6,7,8,9,10,11,12,13], and recent dielectric response techniques have enabled more precise characterization of temperature- and frequency-dependent properties. Yet the vast majority of this work remains confined to macroscopic phenomenology and empirical correlations [14,15,16,17]. Although a few molecular simulations have explored oil–water interactions at the atomistic level, a clear, quantitative linkage between macroscopic dielectric degradation and the spatial distribution, aggregation dynamics, and evolution of water clusters, especially under subzero conditions, has not been established [18,19,20,21]. In particular, the precise role of water aggregation in the degradation of transformer oil remains insufficiently understood. Previous studies have shown that hydrogen-bond interactions promote the aggregation of water molecules into clusters, while moisture accumulation at oil–insulation interfaces can increase interfacial effects and distort the local electric field [22,23]. On the other hand, first-principles calculations have suggested that water impurities can introduce localized electronic states that act as charge traps and facilitate electron capture, thereby potentially affecting charge transport and the electrical breakdown process [16,24,25]. These different microscopic perspectives highlight the need for a mechanistic and multiscale description that connects moisture evolution from dissolution and nucleation to macroscopic clustering with the deterioration of breakdown performance.

In this article, the present study aims to bridge this gap by systematically combining macroscopic electrical measurements, including conductivity and AC breakdown tests, with molecular dynamics simulations to investigate how temperature governs water aggregation and in turn modulates charge carrier behavior and breakdown pathways in moist transformer oil. Our principal findings reveal a non-monotonic temperature dependence, with a distinct offset between conductivity and breakdown extrema, and demonstrate that the transition from dispersed water molecules to stable nanoscale clusters intensifies local field distortion and interfacial polarization, ultimately providing a mechanistic explanation for the anomalous low-temperature insulation failure. This work offers a theoretical foundation for improved insulation design, condition assessment, and moisture management strategies for transformers operating in cold environments.

## 2. Experimental Methods

### 2.1. Sample Preparation and Moisture Control

A mineral transformer oil was selected as the base oil. To obtain samples with a moisture content of 20 mg/kg, a combination of vacuum drying and controlled water addition was employed. The vacuum drying process was performed at a pressure lower than 100 Pa and maintained for at least 24 h to ensure sufficient moisture removal. After drying, deionized water was injected into the oil using a microsyringe and the mixture was stirred continuously for 2 h to achieve a homogeneous moisture distribution. The prepared samples were subsequently stored in sealed glass containers for 24 h prior to testing to allow moisture equilibration. During the moisture conditioning process, a coulometric Karl Fischer titrator with an accuracy better than ±1 mg/kg was used to monitor the moisture content of the transformer oil in real time, ensuring that each sample reached the target moisture level in accordance with IEC 60814 [26].

To further verify the uniformity of the moisture distribution, four independent oil samples were collected from each prepared oil sample for moisture-content measurement. Before each sampling, the container was thoroughly shaken to ensure sufficient mixing and to avoid repeatedly sampling oil from the same local region. The measured moisture contents showed good consistency among the four measurements for each prepared sample. For conciseness, the results of one representative sample are presented in Table 1, where all four measurements were close to the target value of 20 mg/kg, confirming the satisfactory uniformity of the moisture distribution in the prepared oil.

Prior to testing, the oil samples were poured into test cells and conditioned at target temperatures. Electrical measurements were conducted only after thermal equilibrium was reached, as confirmed by built-in temperature sensors, to minimize the influence of temperature fluctuations.

### 2.2. Bulk Conductivity Measurement

The bulk conductivity of the oil samples was measured under a direct current (DC) electric field using a YTC339 dielectric conductivity tester (Shanghai Xuji Electrical Co., Ltd., Shanghai, China) equipped with a three-electrode oil cell. The measurement was conducted in accordance with IEC 60247:2004 [27], while the three-electrode oil cell conformed to the requirements of GB/T 5654–2007 [28].

The oil cell was placed in a temperature-controlled chamber, and the oil temperature was continuously monitored. After reaching the target temperature, the system was maintained under isothermal conditions for at least 10 min to ensure thermal equilibrium. A DC voltage of 500 V was then applied, and the conductivity value was recorded once the reading stabilized. Each measurement was repeated at least 3–5 times, and the average value was taken as the final result. The selected test voltage of 500 V ensured a measurable conduction current while avoiding possible electrohydrodynamic effects and charge injection phenomena that could distort the intrinsic conductivity of the oil. The shielding electrode configuration effectively minimized surface leakage currents and external electromagnetic interference. The uncertainty of temperature control during measurements was maintained within ±0.5 °C.

### 2.3. AC Breakdown Test

The AC breakdown tests were conducted in a low-temperature chamber capable of reaching −40 °C. The oil samples and spherical cap electrode system were placed in the chamber and conditioned at predefined temperatures (−25 °C, −20 °C, −15 °C, −10 °C, −5 °C, 0 °C, 5 °C, 10 °C, 20 °C, and 30 °C).

After thermal stabilization, AC voltage was applied at power frequency with a constant ramp rate of 1 kV/min in accordance with IEC 60156 until breakdown occurred [29]. The electrode gap *d* was set to 1 mm. The breakdown voltage *U*_BD_ was recorded, and the breakdown strength *E*_BD_ was calculated as *E*_BD_ = *U*_BD_/*d*. Each test condition was repeated at least five times, and the mean value and standard deviation were obtained. To minimize the influence of bubbles and impurities, the oil samples were allowed to stand prior to testing, and mechanical disturbances were avoided during voltage application. To improve statistical reliability, breakdown data were further analyzed using Weibull statistical methods. The characteristic breakdown strength and data dispersion were evaluated based on the two-parameter Weibull distribution. Such analysis reduces the influence of random fluctuations inherent to liquid dielectric breakdown and provides a more representative description of insulation performance. The low-temperature breakdown test platform for liquid dielectrics is shown in Figure 1.

## 3. Simulation Methods

### 3.1. Model Construction

Mineral oil is a complex mixture composed of various hydrocarbons, mainly including paraffins, naphthenes, and aromatic compounds. To characterize the structural evolution of moisture in oil at the molecular scale under different temperatures, an oil–water mixed molecular system was constructed using Materials Studio 2023.

Representative oil molecules were selected and combined in appropriate proportions to approximate the composition and density of actual mineral transformer oil. A certain number of water molecules were introduced into the system, corresponding to an initial moisture concentration of c_oil,0_ = 1.05 kg·m^−3^. It should be noted that the moisture concentration used in the MD simulations (1.05 kg·m^−3^) is significantly higher than that employed in the experiments (approximately 20 mg/kg, equivalent to about 20–30 ppm). Such a higher concentration was intentionally adopted to accelerate the formation and evolution of water clusters within the accessible simulation size and timescale. Therefore, the simulation results are intended to reveal the qualitative microscopic mechanism of moisture aggregation under different temperatures rather than to reproduce the exact moisture level in practical transformer oil. Nevertheless, the temperature-dependent aggregation behavior observed in the simulations provides useful insight into the physical processes that may also occur at lower moisture concentrations. The same initial moisture content was maintained across all temperature conditions to ensure comparability.

The constructed multicomponent system was subjected to periodic boundary conditions (PBC) to generate the initial simulation cell for subsequent molecular dynamics calculations. The composition and content of the mineral oil model compounds are shown in Table 2, and the diagram of the oil–water mixture molecular model constructed using Materials Studio is shown in Figure 2. Representative molecules were used to model each hydrocarbon class.

The condensed-phase optimized molecular potentials for atomistic simulation studies (COMPASS) force field was selected because it has been extensively validated for condensed-phase organic systems and has demonstrated good accuracy in reproducing thermophysical properties of hydrocarbon mixtures and hydrogen-bonded systems. Previous studies have shown that COMPASS can reliably characterize intermolecular interactions between transformer oil molecules and moisture, making it suitable for investigating temperature-dependent aggregation behavior. The intermolecular interactions were described using the COMPASS force field. Non-bonded interactions were modeled by combining Coulombic potential and Lennard–Jones (LJ) potential:

(1) $$g_{\mathrm{coul}}=\sum_{i<j}\frac{q_{i}q_{j}}{4\pi \varepsilon_{0}r_{\mathrm{ij}}},$$

(2) $$U_{\mathrm{LJ}}=4\varepsilon [{\left(\frac{\sigma }{r_{\mathrm{ij}}}\right)}^{12}-{\left(\frac{\sigma }{r_{\mathrm{ij}}}\right)}^{6}],r_{\mathrm{ij}}<r_{c},$$

where *g*_coul_ is the Coulombic potential, J; *q*_i_ is the partial charge of atoms *i*, e; *r*_ij_ is the distance between atoms *i* and *j*, Å; *U*_LJ_ is the Lennard–Jones (LJ) potential, J; *ε* is the potential well depth, kJ·mol^−1^; and *σ* is the characteristic distance at which the potential becomes zero, Å; *r*_c_ is the cutoff distance, Å.

### 3.2. Simulation Parameters and Procedure

Simulations were carried out at ten temperature points: −25 °C, −20 °C, −15 °C, −10 °C, −5 °C, 0 °C, 5 °C, 10 °C, 20 °C, and 30 °C.

The MD simulation process consists of five main steps, as shown in Figure 3.

1. Model construction

A molecular model of mineral insulating oil was established, and water molecules with specified concentrations were introduced. Appropriate force field parameters were selected for different simulation systems.

2. Energy minimization

Geometry optimization of the initial configuration was performed using the Smart algorithm, with an energy convergence criterion of 10 kcal·mol^−1^ and a maximum of 10,000 iterations.

3. Pre-equilibration (annealing)

Annealing was conducted to obtain a stable and well-relaxed structure. For the nucleation system, simulations were performed under both number–volume–temperature (NVT) and number–pressure–temperature (NPT) ensembles to eliminate internal stresses. The system temperature was cycled from 258 K to 458 K and back to 258 K with an interval of 10 K, and each cycle was repeated five times. After annealing, system energy and density stabilized, and unstable configurations were eliminated.

4. Dynamic simulation

The leapfrog algorithm was employed for time integration with a time step of 1 fs. The Nose–Hoover thermostat was used to control temperature, and the pressure was maintained at 0.1 MPa. The convergence criterion required fluctuations in energy, temperature, density, and simulation box size to be within 5%. The total simulation time was 3000 ps.

5. Data analysis

Trajectory data were extracted for post-processing, and the microscopic dynamic mechanisms and thermodynamic properties were analyzed.

## 4. Results and Discussion

### 4.1. Macroscopic Electrical Performance Under Low-Temperature Conditions

Moisture and temperature are key factors affecting the dielectric properties of transformer oil. To investigate the differences in electrical responses under low-temperature moist conditions, mineral insulating oil with a moisture content of 20 mg/kg was selected, and its bulk conductivity and AC breakdown strength were systematically measured at different temperatures. The bulk conductivity of transformer oil with a water content of 20 mg/kg at different temperatures is shown in Figure 4.

As shown in Figure 4, the bulk conductivity of the oil exhibits a pronounced non-monotonic dependence on temperature. As the temperature decreases from 30 °C to approximately −5 °C, the conductivity gradually increases and reaches a peak near −5 °C. However, when the temperature further decreases to −10 °C and below, the conductivity begins to decline, forming a typical “single-peak” temperature response. This behavior deviates significantly from the classical thermally activated conduction mechanism, in which conductivity monotonically decreases with temperature. It indicates that the conduction process in moist transformer oil at low temperatures is not solely governed by charge carrier mobility, but is strongly influenced by the physical state and distribution of moisture.

In contrast, the variation in AC breakdown strength with temperature, shown in Figure 5, exhibits a more direct and pronounced response. As the temperature decreases from 30 °C into the low-temperature range (approximately −10 °C to −20 °C), the breakdown strength rapidly decreases and reaches a minimum around −10 °C, corresponding to the most unfavorable operating condition. With further temperature increase, the breakdown strength recovers rapidly within the range of 0–20 °C, while the increasing trend gradually becomes moderate at higher temperatures. These results indicate that the breakdown process is highly sensitive to temperature under low-temperature conditions and that a distinct “weakest temperature window” exists.

### 4.2. Nucleation and Aggregation Behavior of Moisture in Transformer Oil

To characterize the intermolecular interactions and spatial correlations between water molecules, the radial distribution function (RDF) of oxygen–oxygen pairs, *g*_O-O_(*r*), was calculated:

(3) $$g_{\left(O-O\right)}\left(r\right)=\frac{1}{4\pi \rho Nr^{2}}\sum_{i\neq j}\delta (r-r_{\mathrm{ij}}),$$

where *ρ* is the number density of oxygen atoms; *N* is the total number of particles; *r*_ij_ denotes the separation between two particles *i* and *j*; *δ*(*r*) is the Dirac delta function that selects pairs with interparticle distance exactly equal to *r*. By analyzing the position, height, and width of the first coordination peak, as well as the intermediate-range decay behavior, the transition of water molecules from a dispersed state to an aggregated state can be identified.

Figure 6 presents the RDF results at different temperatures. The first coordination peak is consistently located at approximately *r* ≈ 0.275 nm, indicating that the characteristic short-range hydrogen-bond geometry between water molecules remains essentially unchanged over the studied temperature range. However, the peak height, width, and decay characteristics exhibit clear temperature dependence. In particular, the first peak reaches its maximum near 0 °C, indicating the highest probability of nearest-neighbor coordination at this temperature. As the temperature deviates from 0 °C toward either higher (30 °C) or lower (−20 °C) values, the peak height decreases, whereas the peak becomes sharper upon cooling, indicating reduced configurational fluctuations and a more concentrated O-O distance distribution. These changes provide molecular-level evidence for enhanced local ordering and moisture aggregation under low-temperature conditions.

To quantitatively characterize the aggregation degree of moisture, cluster analysis based on geometric distance criteria was performed. Two water molecules were considered to belong to the same cluster if their oxygen–oxygen distance satisfies:

(4) $$r_{\left(O-O\right)}\leq r_{c},$$

where *r*_(O-O)_ is the oxygen–oxygen distance; *r*_c_ is the cutoff distance.

The cutoff distance *r*_c_ was selected near the first minimum of the RDF, corresponding to the boundary of the first coordination shell.

Based on this criterion, the cluster size *N*_k_(*t*) was calculated for each frame, and the maximum cluster size was defined as:

(5) $$N_{\max}(t)=\max\{N_{k}(t)\},$$

where *N*_max_(*t*) is the maximum cluster size; *N*_k_(*t*) is the cluster size at every frame.

Time-averaged statistics of *N*_max_(*t*) were used to evaluate the dominant cluster size and its stability at different temperatures. Furthermore, according to the Yasuoka–Matsumoto nucleation criterion, a threshold cluster size *n* = 17 was adopted to distinguish transient small clusters from stable liquid-like aggregates. Thus, *N*_max_ < 17 represents predominantly transient aggregation, whereas *N*_max_ ≥ 17 indicates the formation of a stable liquid-like water aggregate. This criterion provides a quantitative basis for identifying the transition from molecular dispersion to nucleation and subsequent stable aggregation.

As shown in Figure 7, the maximum cluster size *N*_max_ increases significantly with decreasing temperature, while its temporal fluctuations decrease and the persistence of the clusters increases. Combined with the critical cluster size of *n* = 17, these results provide a quantitative criterion for distinguishing the nucleation and stable-aggregation regimes. Near 0 °C, the system exhibits the strongest short-range structural correlation, as indicated by the maximum O-O RDF peak, suggesting that this temperature favors the initial nucleation of water-rich regions. With further cooling, *N*_max_ progressively exceeds the critical size and continues to increase, while its fluctuations become smaller, indicating that the initially formed nuclei grow and evolve toward increasingly stable large-scale aggregates. Therefore, the MD results reveal not only the existence of moisture aggregation but also its temperature-dependent structural transition from transient molecular association to stable cluster formation.

Overall, this process follows a two-stage dynamic pathway: first, near 0 °C, optimal nucleation occurs, forming locally ordered hydrogen-bond networks; subsequently, as the temperature further decreases, these nuclei grow and coalesce into larger clusters driven by reduced solubility.

### 4.3. Unified Mechanism Linking Microscopic Moisture Behavior with Electrical Properties

The simulations in Section 4.2 reveal that the structural evolution of moisture in oil follows a staged transition from dispersion to nucleation and finally to stable aggregation. This evolution corresponds closely to the key temperature points observed in the macroscopic electrical characteristics. The stages of the structural evolution of moisture in oil with temperature are shown in Figure 8.

At high temperatures (30 °C to 0 °C), water molecules are primarily dispersed or form transient small clusters. As temperature decreases, intermolecular interactions strengthen, leading to increased local enrichment and interfacial area. This is likely associated with enhanced interfacial polarization and charge accumulation, resulting in an increase in apparent conductivity.

Near 0 °C, the system reaches a transition state characterized by the strongest structural correlation. The number of interfaces and polarization effects reach a maximum, which is likely to contribute to the peak conductivity.

In the temperature range of 0 °C to −5 °C, water molecules begin transitioning into more stable clusters. Although aggregation progresses, interfacial polarization remains dominant, which is likely to lead to the observed conductivity peak near −5 °C.

When the temperature further decreases to around −10 °C, stable droplet-like clusters dominate. These clusters introduce strong dielectric discontinuities, causing severe local electric field distortion and charge accumulation. Combined with the increased cluster size acting as “soft defects,” the breakdown threshold is significantly reduced, resulting in the observed minimum in breakdown strength.

At even lower temperatures, molecular motion is further restricted, and both charge transport and interfacial dynamics are suppressed. Consequently, conductivity decreases, and breakdown deterioration does not continue to intensify.

These simulation results provide further insight into the structure–property relationship of moisture in transformer oil. The increase in the intensity of the O–O correlation and the progressive formation of water aggregates indicate that decreasing temperature changes the moisture from a relatively dispersed state to a more spatially heterogeneous configuration. This structural evolution increases the extent of oil–water interfaces within the system, thereby enhancing dielectric heterogeneity and interfacial polarization. Such a microscopic change is consistent with the experimentally observed variation in bulk conductivity. At lower temperatures, the formation of larger and more stable water clusters further increases the spatial nonuniformity of the dielectric environment. Under an applied electric field, these regions can therefore act as local dielectric discontinuities, favoring local electric field distortion and charge accumulation. This provides a microscopic interpretation of the experimentally observed deterioration in AC breakdown strength. The local field effect can be understood from the strong dielectric contrast between the water-rich regions and the surrounding oil matrix. As water molecules become concentrated into larger clusters, the resulting heterogeneous regions provide preferential locations for electric field redistribution. The larger and more stable clusters at lower temperatures therefore have a greater potential to produce local field enhancement and charge accumulation, which can facilitate the initiation of electrical breakdown.

In summary, both the conductivity peak (−5 °C) and the breakdown minimum (−10 °C) originate from the same microscopic mechanism: temperature-induced nucleation and aggregation of moisture. However, they correspond to different dominant effects: interfacial polarization for conductivity and local field distortion for breakdown. This leads to the observed offset between the two phenomena.

The MD simulation results provide a molecular-level interpretation of the experimentally observed temperature-dependent electrical behavior. The simulated transition of water molecules from a dispersed state to nucleated aggregates and subsequently to stable large-scale clusters is consistent with the temperature-dependent variations observed experimentally. Specifically, the formation of water aggregates increases the oil–water interfacial area and enhances interfacial polarization, providing a microscopic explanation for the conductivity peak. With further cooling, the formation of stable water clusters introduces local dielectric inhomogeneities and causes local electric field distortion, which is consistent with the experimentally observed deterioration in breakdown strength. Thus, the MD simulations corroborate the experimental results by revealing the microscopic moisture evolution underlying the observed electrical responses.

It should be noted that the moisture concentration adopted in the MD simulations is considerably higher than that used in the experiments and is not intended to quantitatively reproduce practical transformer operating conditions. The elevated moisture content was introduced to facilitate the observation of moisture nucleation and aggregation phenomena within the accessible simulation size and timescale. Therefore, the simulation results should be interpreted as providing qualitative insight into the temperature-dependent evolution of moisture structures and their potential influence on electrical properties, rather than a direct quantitative prediction of conductivity or breakdown strength.

## 5. Conclusions

This study systematically investigates the degradation behavior and underlying mechanisms of transformer oil under low-temperature moist conditions by combining macroscopic electrical measurements with molecular dynamics simulations. The results show that within the low-temperature range (−30 °C to 10 °C), the bulk conductivity exhibits a peak near −5 °C, while the AC breakdown strength shows a pronounced minimum around −10 °C, demonstrating a non-monotonic and offset temperature response. This phenomenon originates from the temperature-dependent evolution of moisture in oil, transitioning from a dispersed state at high temperatures to nucleation near 0 °C, and eventually forming stable large-scale clusters at lower temperatures. The conductivity peak is likely associated with enhanced interfacial polarization arising from the increased oil–water interfacial area during the nucleation stage, whereas the breakdown minimum is directly related to stable water clusters acting as dielectric “soft defects,” which induce local electric field distortion and reduce the breakdown threshold.

A comprehensive correlation framework linking temperature, microscopic moisture aggregation, and macroscopic electrical properties is established. The findings not only deepen the understanding of dielectric behavior in oil–water systems under complex conditions but also provide insight into the influence of low-temperature moisture evolution on the electrical properties of transformer oil and may contribute to future studies on insulation behavior in transformer oil–paper systems operating in cold environments. In particular, the identification of the weakest temperature window and the role of moisture clustering offer valuable guidance for moisture control strategies and reliability enhancement.

## Figures and Tables

**Figure 1:**
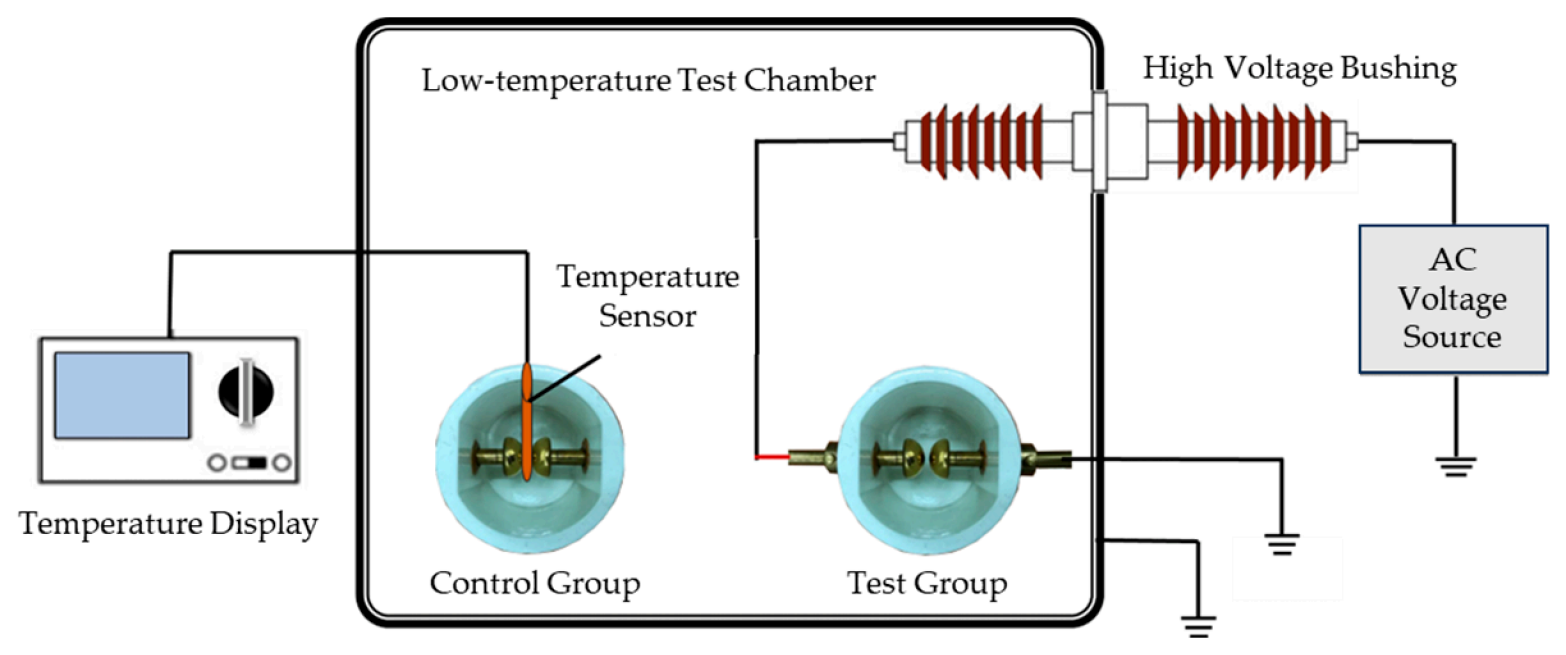
Low-temperature breakdown test platform for liquid dielectrics.

**Figure 2:**
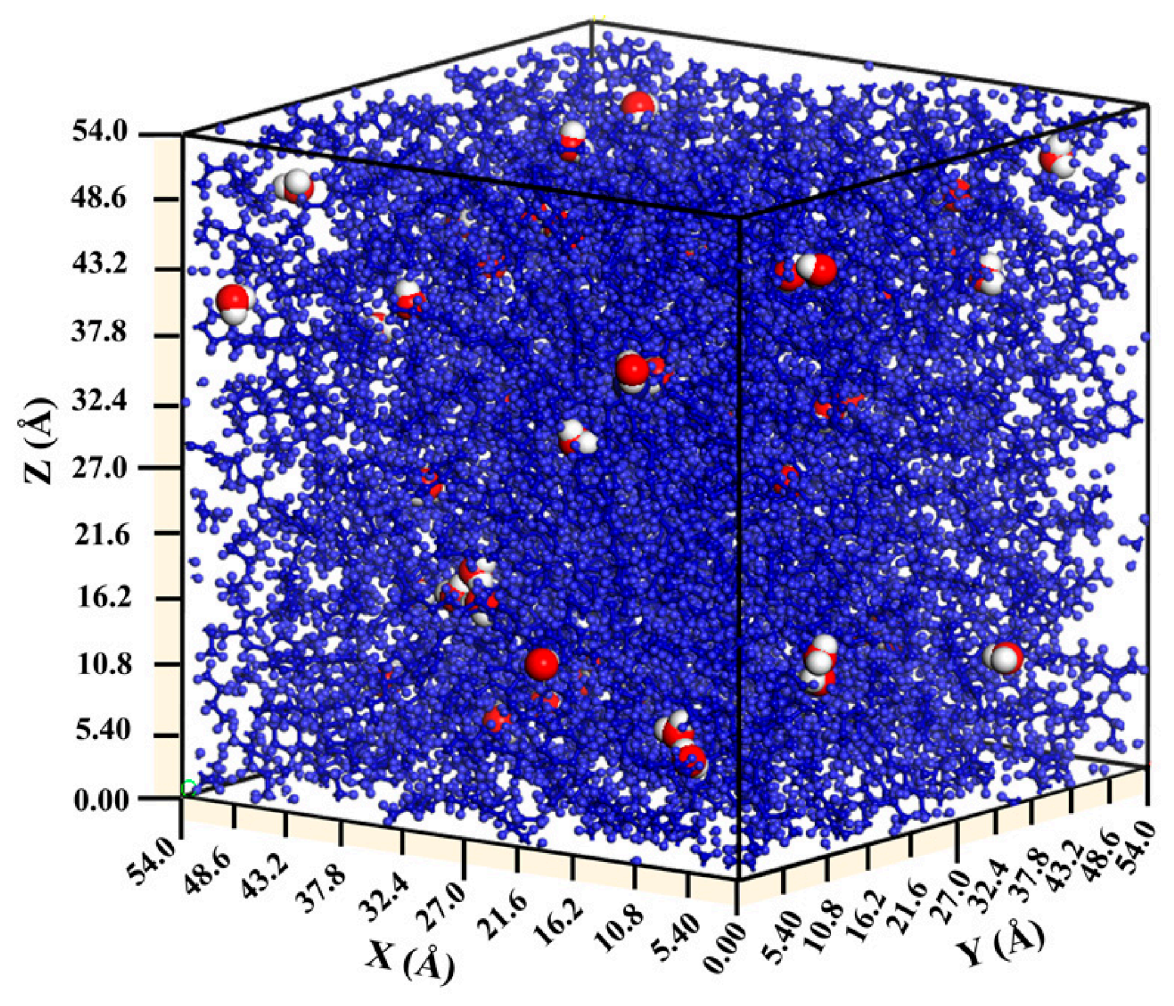
The diagram of oil–water mixture compound model. (C: 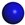; H: 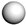; O: 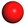).

**Figure 3:**
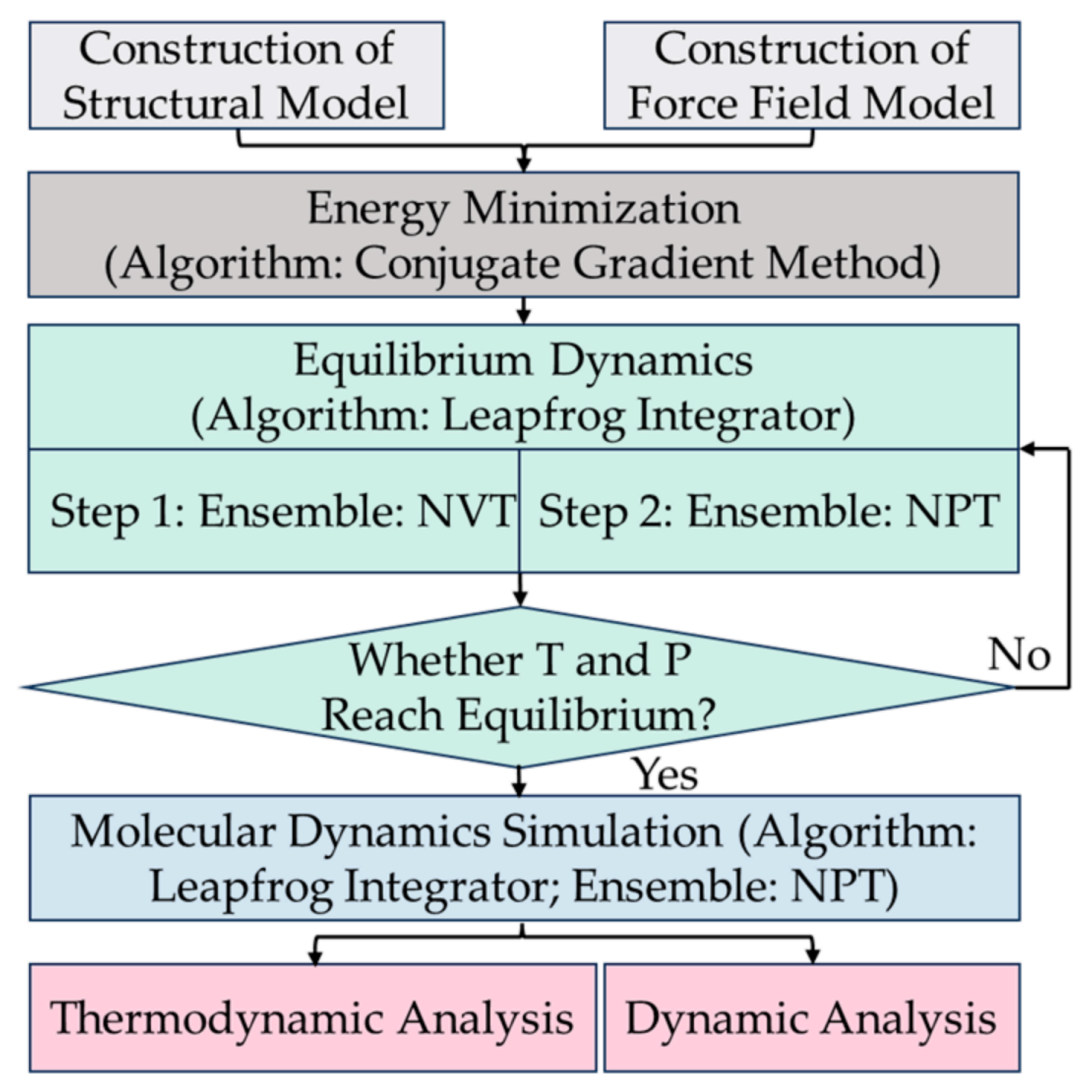
The algorithm process of MD simulation.

**Figure 4:**
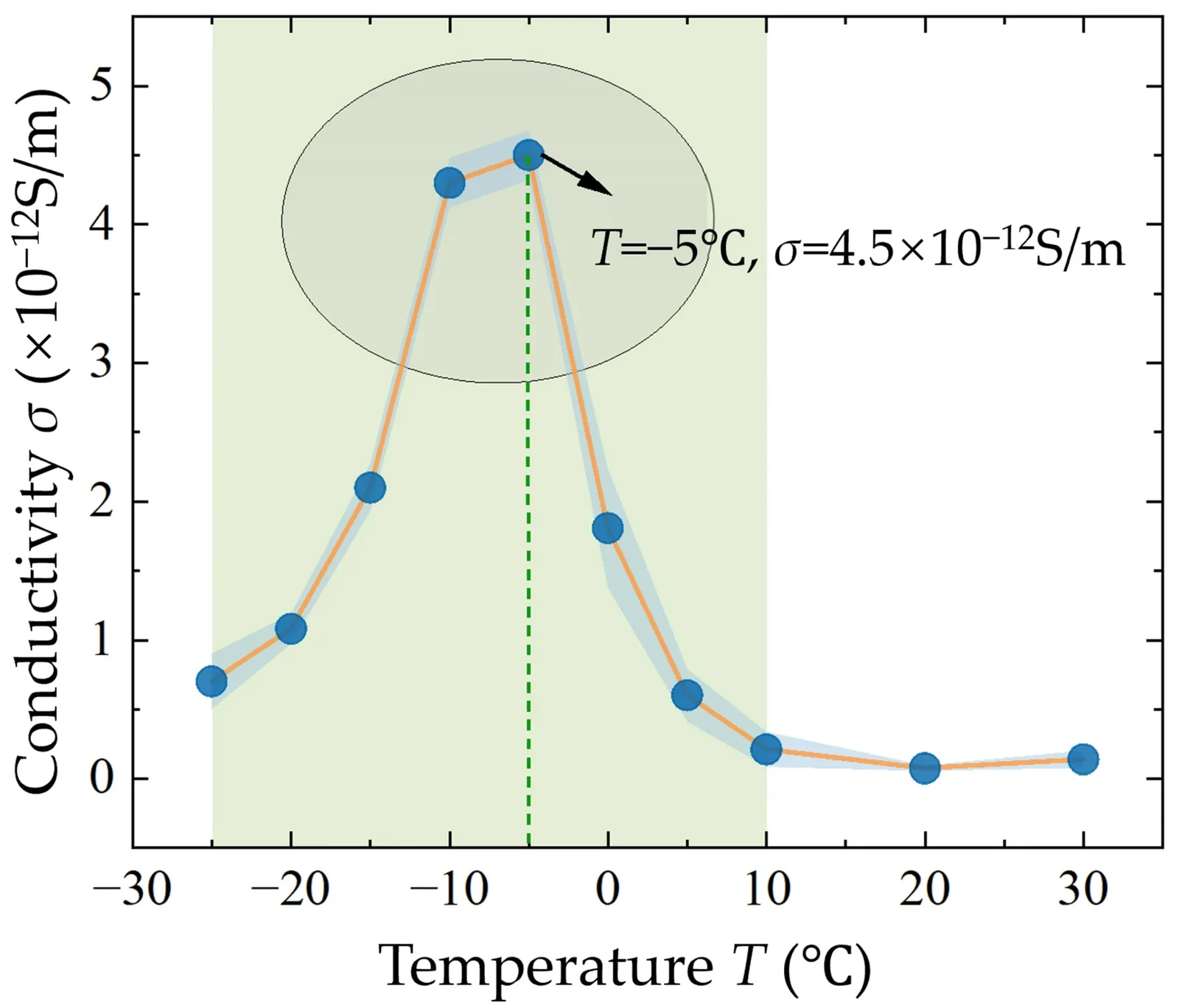
Bulk conductivity of transformer oil with a water content of 20 mg/kg at different temperatures.

**Figure 5:**
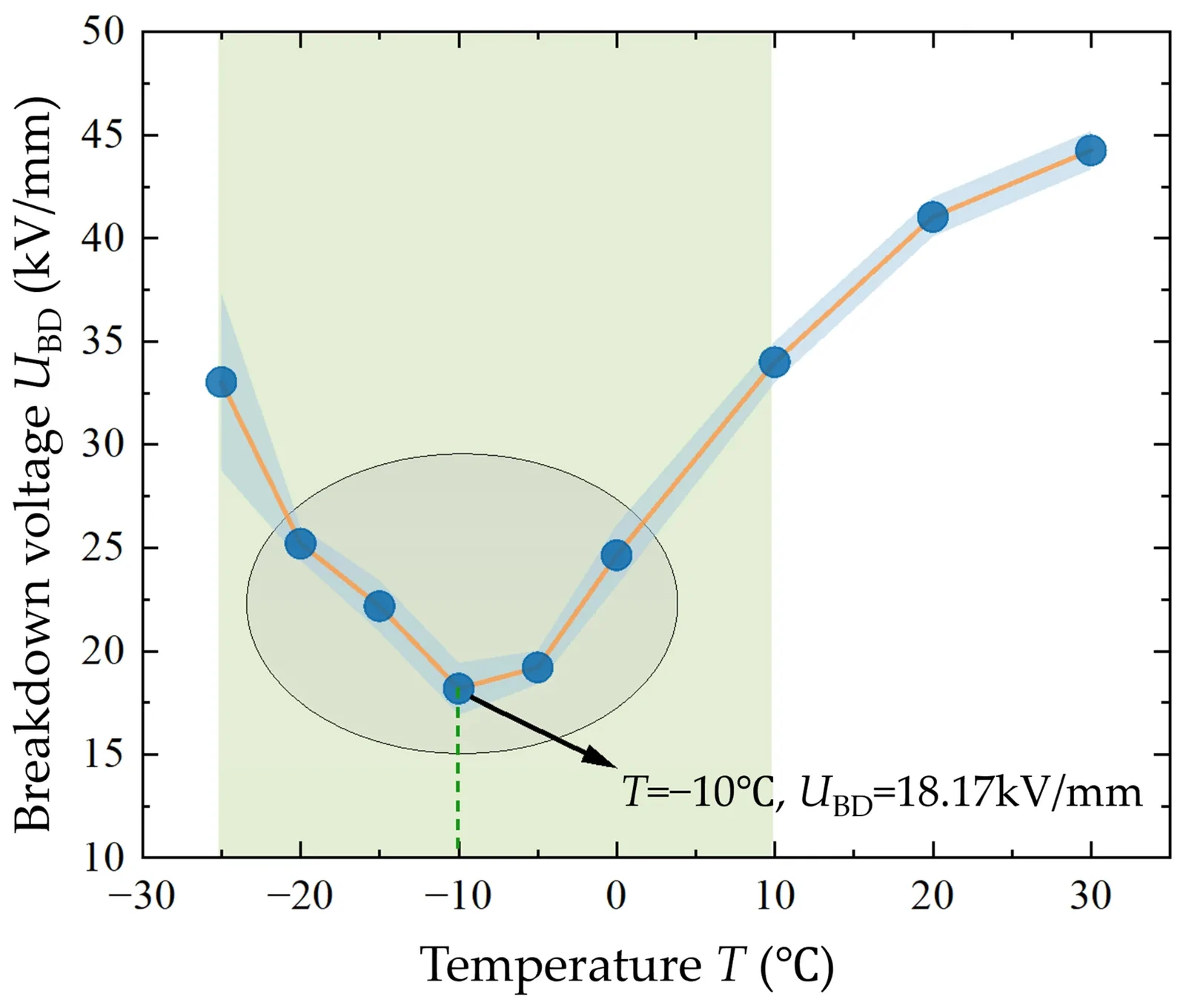
AC breakdown strength of insulating oil with a water content of 30 ppm at different temperatures.

**Figure 6:**
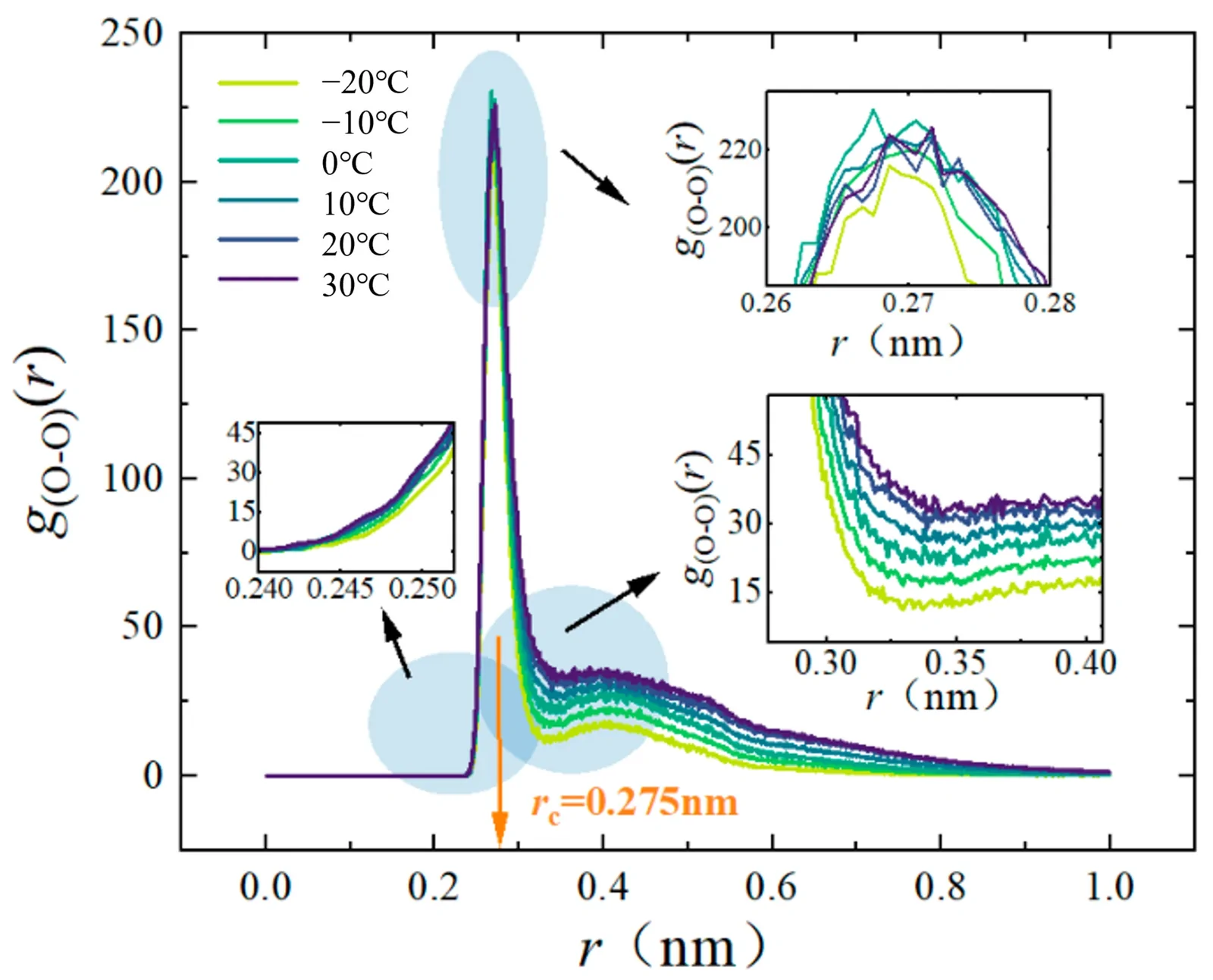
Water–water radial distribution function *g*_O-O_(*r*) in transformer oil at different temperatures.

**Figure 7:**
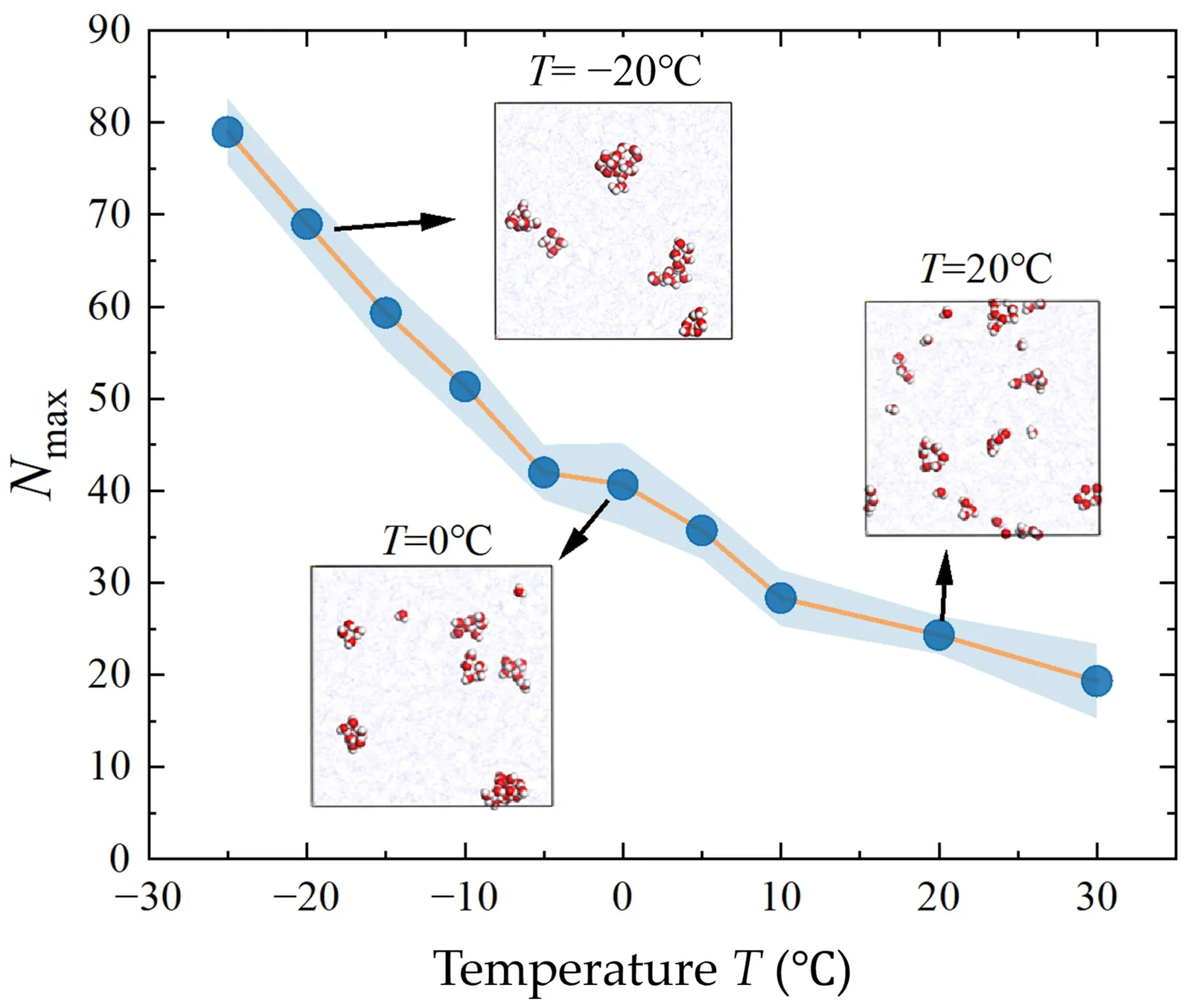
The variation in *N*_max_ at different temperatures and a schematic diagram of typical aggregation configurations of water in transformer oil.

**Figure 8:**
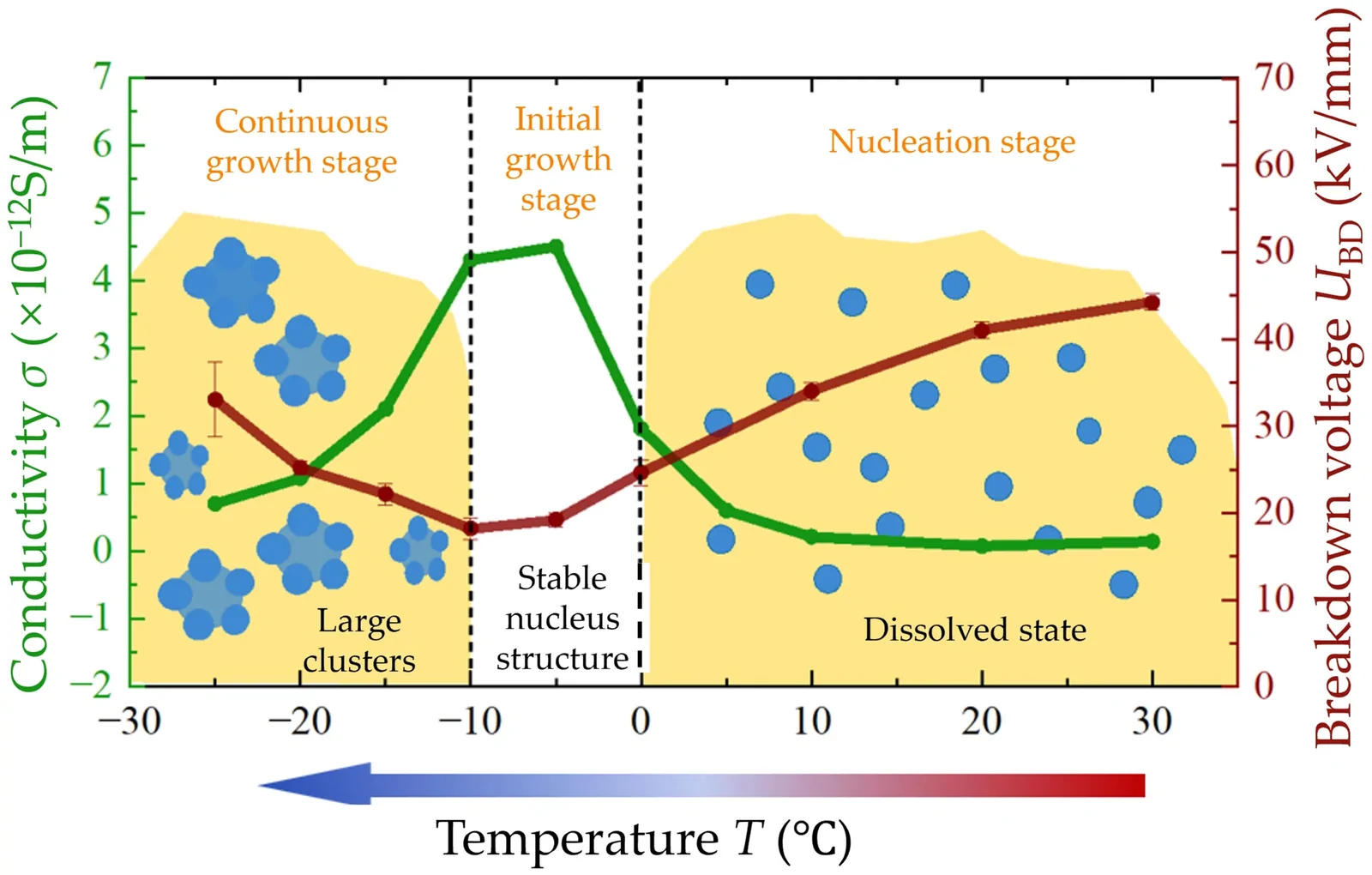
The stages of the structural evolution of moisture in oil with temperature.

**Table 1:** Moisture content obtained from four independent measurements of a representative prepared oil sample.

Measurement No.	1	2	3	4	Mean ± SD
Moisture content (mg/kg)	20.1	19.8	20.2	19.9	20.0 ± 0.18

**Table 2:** The composition and content of mineral oil model compounds.

Composition	Alkane	Cycloalkane				Aromatic Hydrocarbon
		Monocyclic Alkane	Bicyclic Alkane	Tricyclic Alkane	Tetracyclic Alkane	
Content (wt%)	11.6	15.5	28.5	23.3	9.4	8.3

## Data Availability

The original contributions presented in this study are included in the article. Further inquiries can be directed to the corresponding author.

## Abbreviations

The following abbreviations are used in this manuscript:

AC	Alternating current
MD	Molecular dynamics
DC	Direct current
PBC	Periodic boundary conditions
COMPASS	Condensed-phase optimized molecular potentials for atomistic simulation studies
NVT	Number–volume–temperature
NPT	Number–pressure–temperature
LJ	Lennard–Jones
RDF	Radial distribution function
